# Predicting COVID-19 cases using bidirectional LSTM on multivariate time series

**DOI:** 10.1007/s11356-021-14286-7

**Published:** 2021-05-27

**Authors:** Ahmed Ben Said, Abdelkarim Erradi, Hussein Ahmed Aly, Abdelmonem Mohamed

**Affiliations:** Computer Science and Engineering Department, College of Engineering, Qatar University, 2713 Doha, Qatar

**Keywords:** COVID-19, Cumulative cases, Bi-LSTM, Clustering

## Abstract

To assist policymakers in making adequate decisions to stop the spread of the COVID-19 pandemic, accurate forecasting of the disease propagation is of paramount importance. This paper presents a deep learning approach to forecast the cumulative number of COVID-19 cases using bidirectional Long Short-Term Memory (Bi-LSTM) network applied to multivariate time series. Unlike other forecasting techniques, our proposed approach first groups the countries having similar demographic and socioeconomic aspects and health sector indicators using K-means clustering algorithm. The cumulative case data of the clustered countries enriched with data related to the lockdown measures are fed to the bidirectional LSTM to train the forecasting model. We validate the effectiveness of the proposed approach by studying the disease outbreak in Qatar and the proposed model prediction from December 1st until December 31st, 2020. The quantitative evaluation shows that the proposed technique outperforms state-of-art forecasting approaches.

## Introduction

In December 2019, Wuhan, the capital of Central China’s Hubei province, with 11 million population, has witnessed the outbreak of a new coronavirus (COVID-19) (Yang et al. 2020; Chauhan 2020). The virus has propagated in China then all over the world. On the 11th of March 2020, with more than 280k cases and more than 4000 deaths worldwide, it has been declared as a global pandemic by the World Health Organization (WHO) (WHO 2020). Within few months, the number of cases has exponentially grown to more than 17 million and more than 670k deaths by the end of July 2020. By March 2021, the total cases reached more than 117 million cases and unfortunately 2.6 million deaths. Since the emergency approval of multiple vaccine products, countries worldwide are in a rush to acquire the vials and start vaccination campaigns. By the beginning of March 2021, the cumulative COVID-19 vaccination doses administered per 100 people reached 27.3 in the USA, 35.1 in the UK, and 65.1 in UAE.^1^ Worldwide, more than 312 million shots are given. In the UK, the ease of the strict lockdown imposed since September has started On March 8th, all schools reopened with after-school sports allowed. In public spaces, recreation is permitted between two persons. Qatar has witnessed a significant decrease in the number of cases compared to summer 2020 and reached around 200 cases until January 2021. By the end of January, the number of cases has doubled and becomes stable at around 450 cases. The fourth phase of restriction is still mandated while the vaccination campaign is ongoing. By March 7th, 61% of people over 80 have at least received one dose of the vaccine, while 10% of Qatar’s entire adult population have received at least one vaccine dose.

A plethora of research works have been conducted to get better insights into the propagation of the virus and the evolution of the number of cases and deaths while racing against the time to develop an effective vaccine.

In the following section, we focus on recent research efforts that leveraged the power of machine learning and mathematical modeling techniques to study the propagation of COVID-19 and the evolution of the number of cases.

## Literature review

Research works can be categorized according to the adopted technique: machine learning based and mathematical modeling based.

### Machine learning for COVID-19

Since the virus outbreak, researchers from multiple disciplines started looking at the different kinds of resulting data, including the daily cases, cumulative cases, CT-scan, and MRI images of patient lungs. These data are of paramount importance as they enable more in-depth study of the virus from different perspectives.

Saba and Elsheikh (2020) studied the propagation of COVID-19 in Egypt and applied a nonlinear autoregressive artificial neural network to forecast the virus prevalence. The authors modeled the confirmed cases as time series and compared their approach against Auto-Regressive Integrated Moving Average (ARIMA) model. Both techniques are used to forecast the cumulative COVID-19 cases for 10 days (1 to 10 April 2020) using the confirmed cases reported in March. In Petropoulos and Makridakis (2020), the authors applied exponential smoothing approach and conducted five rounds of forecast of cumulative confirmed cases globally starting from the 1st of February until 21st of March 2020. The authors emphasized that forecasts related to the virus outbreak must be an integral part of any decision-making process, particularly in high-risk areas. Indeed, this enables authorities to explore various “what if” scenarios to assess the implication of any decision. Ahmar et al. (Ahmar and del Val 2020) proposed to apply a variety of ARIMA, called SutteARIMA, for short-term forecast of COVID-19 cases in Spain and the impact on the Spanish Market Index (IBEX). Data from February 12 until April 2 are used to train the model to forecast the data from April 3 to 9. The Mean Absolute Percentage Error (MAPE) metric is calculated to assess the fitting accuracy. The findings showed that SutteARIMA outperformed ARIMA model. In Ribeiro et al. (2020), the authors compared six prediction techniques to forecast the cumulative cases in ten Brazilian states: ARIMA, cubist regression, random forest, ridge regression, support vector regression, and stacking-ensemble learning. The prediction is conducted for multiple time horizons: 1 day, 3 days, and 6 days ahead. Chimmula and Zhang (2020) studied the propagation of the virus in Canada using Long Short-Term Memory (LSTM) neural network, known to be efficient with sequential data. The results show that Canada had a linear growth of the number of cases until March 16, 2020, followed by an exponential growth. It has been estimated that the ending point of the outbreak is around June. Maleki et al. (2020) applied TP-SMN-AR, a variation of autoregressive models, to forecast the confirmed and recovered the number of cases worldwide. This prediction is conducted for the period between April 21 until April 30.

The recent advances in deep learning have revolutionized the healthcare industry. Various applications have been implemented and commercialized which incorporated AI-driven component that assists doctors and healthcare providers in achieving accurate diagnosis (Ahmadi et al. 2021; Ahmadi et al. 2021). In the context of COVID-19, multiple research efforts proposed diagnosing COVID-19 using data provided from medical imaging techniques such as MRI and CT-scan. This problem is quite challenging as data are not widely available and require expert knowledge for annotation. Hassantabar et al. (2020) proposed a convolutional neural network (CNN)–based approaches to diagnose and localize infected tissue of COVID-19 patients based on X-ray images of lungs. The first deep learning model is a deep neural network trained on fractal features of the images. The second model is CNN-based trained on images of lungs. Results showed that the CNN-based model achieved 93.2% accuracy outperforming the first model that achieved 83.4% accuracy. He et al. (2021) proposed a novel deep learning architecture trained on 3D CT volume of lungs. The volume is first split into 2D patches and fed into an encoding part for feature extraction. The encoding module is followed by two sub-networks for joint classification and segmentation. The classification part consists of feature embedding, a feature learning module, and a classifier to determine patient severity (severe-non severe). The segmentation part consists of a decoding network that outputs a segmented lung lobe.

Although diagnosing COVID-19 based on medical imaging is a promising area of research, the problem is challenging, prone to data annotation errors, and requires expert knowledge, not to mention the scarcity of the data.

### Mathematical models for COVID-19

Mathematical models of infectious disease have also been applied in attempt to obtain better insight into the virus outbreak. Kuniya (2020) used the SEIR model to predict the epidemic peak in Japan from 15 January to 29 February 2020. SEIR provides a mathematical formulation to describe the transmission of a disease from an individual to another. These individuals pass through four states: susceptible (S), exposed (E), infectious (I), and recover (R). The study showed that the basic reproduction number *R*_0_—“the average number of secondary infections produced by a typical case of an infection in a population where everyone is susceptible” Rothman and Lash (2008)—is 2.6 with a 95% confidence interval 2.4–2.8. The SEIR model also showed that the peak would occur on early-middle summer 2020. Furthermore, some epidemiological conclusions are drawn: the intervention has great implications on delaying the epidemic peak. It also must be conducted over a long period to ensure effective reduction of the epidemic size. In Boudrioua and Boudrioua (2021), the authors applied the SIR model to predict Algeria’s daily cases. SIR takes into account the number of susceptible cases (S), the number of infected cases (I), and the number of recovered cases (R). The model showed that the peak was expected on July 24, 2020, at worst and that the disease would disappear between September and November. Roosa et al. (2020) used three phenomenological models: the generalized logistic growth model (Viboud et al. 2016), the Richards model (Richards 1959), and a sub-epidemic wave model (Chowell et al. 2019) for real-time forecast of the COVID-19 cumulative number of confirmed reported cases in Hubei province, China. These models were previously applied to forecast several infectious diseases, including Ebola, SARS, pandemic Influenza, and Dengue. Authors in Gupta et al. (2020) studied the effect of weather on the spread of COVID-19. Using the daily cases in 50 US states between January 1 and April 9, 2020, in addition to temperature and absolute humidity information, the authors identified the vulnerable narrow absolute humidity range. States with absolute humidity between 4 and 6 g/m^3^ have a significant spread with more than ten thousand cases. The findings are used to determine the Indian regions with potential vulnerability to weather-based spread. Ahmadi et al. (2021) investigated the termination time of the outbreak in Iran. Using the single-peak SIR model, COVID-19 is predicted to terminate in June 2020, which is invalid as, by end of February 2021, the country has around 190k cases. The authors addressed this issue using the generalized logistic growth model to estimate the epidemic waves of the virus. Furthermore, the impact of travel between cities on the number of cases has been addressed. The findings showed travel between Tehran and other major cities resulted in a higher risk of infection, reaching more than 100 per day, hence the importance of imposing effective restrictions to control the outbreak.

It is widely known that lockdown measures, e.g., restriction on gathering, school and workplace closing, public transport shutdown, and international travel controls, are needed for halting the spread of the virus. Atalan (2020) conducted data analysis and showed evidence that lockdown can contribute in suppressing COVID-19 pandemic. Dawoud (2021) emphasized on the importance of preventive measures, including social distancing and mask usage for an efficient lockdown exit strategy. Sahoo and Sapra (2020) conducted a data-driven approach to analyze the effect of lockdown in India. The authors showed that after 6 weeks of lockdown, the infection rate reached three times lower compared to the initial one. Hence, the lockdown measures are quintessential to manage such pandemic. However, these measures are rarely considered when forecasting COVID-19 daily or cumulative cases. Furthermore, most COVID forecast methods typically rely on limited data of a single country. Yet countries having common demographic and socio-economic properties and similar health sector indicators can exhibit similar pandemic patterns. Our contribution consists of first grouping countries having similar demographic and socio-economic properties and health sector indicators, then using COVID-19 data from each cluster to build the prediction model. This yields a richer dataset for training. Furthermore, we propose a deep learning–based forecasting approach based on bidirectional LSTM (Bi-LSTM). This type of neural network not only relies on the past data to predict the future, but it also enables learning from the future to predict the past. By adopting such a learning framework, Bi-LSTM provides better understanding of the learning context (Schuster and Paliwal 1997). Additionally, to train our Bi-LSTM-based model, we rely on multivariate time series consisting of the cumulative daily number of cases and time series describing the lockdown measures: the school closing, workspace closing, restriction on gathering, public transport closing, and international travel controls. The proposed Bi-LSTM on multivariate time series allows multiple dependent time series to be modeled together to account for the correlations cross and within the series capturing variables changing simultaneously over time.

## Cumulative cases prediction approach using Bi-LSTM on multivariate time series

**Fig. 1:**
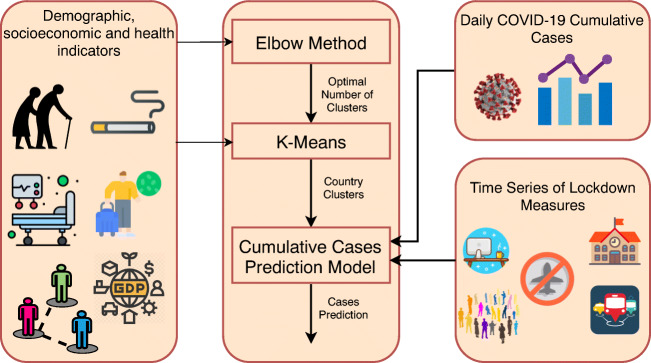
Overview of the proposed prediction approach of daily cumulative cases of COVID-19 using Bi-LSTM on multivariate time series

We depict in Fig. 1 the overall approach to predict the daily cumulative cases of COVID-19. First, we collect data describing the demographic and socioeconomic properties and health sector of countries worldwide. These data are clustered to identify the group of countries having similar properties. We first apply the elbow method to determine the optimal number of clusters, which is then fed as an input parameter to the K-means algorithm. Next, given a particular country, we identify its cluster. Multivariate time series are then constructed consisting of daily cumulative cases of all countries belonging to the cluster in addition to time series describing the level of lockdown measures associated with travel control (border closing), school closing, workplace closing, public transport shutdown, and public gathering ban. The multivariate time series are used to train a deep learning Bi-LSTM network to forecast future cumulative number of cases. It is worthwhile to mention that this approach is applicable for any country to forecast its daily cumulative COVID-19 cases.

### Clustering countries based on demographic, socioeconomic, and health sector indicators

We describe in this section the demographic, socioeconomic, and health sector indicators used to cluster countries. Then, we present the technique used to group these countries. This yields a richer dataset for training COVID-19 cumulative cases prediction model per countries’ cluster.

#### Demographic, socioeconomic, and health sector indicators data

These data have been collected from the Department of Economic and Social Affairs of the United Nations and the Organization for Economic Cooperation and Development. The data include:

- Median age per country.
- Population percentage of age groups per 5-year interval, e.g., 4–9 years and 10–14 years.
- Country population and density.
- The percentage of urban population.
- Gross domestic product (GDP) per capita.
- The number of hospitals per 1000 people.
- Death rate from lung diseases per 100k people for female and male.

#### Countries clustering

To discover countries with similar characteristics, we applied the K-means clustering algorithm (Jain 2010; B Said et al. 2013) to identify similar members among the data points. Let *X* = {*x*_1_,*x*_2_,...,*x*_*n*_} be the set of d-dimensional points we seek to cluster into *K* clusters. In other words, we attempt to assign each *x*_*i*_, *i* = 1,...,*n* to a cluster *c*_*k*_, *k* = 1,...,*K*. K-means partitions the data such that the squared error between the mean of a cluster and the data points, members of the clusters, is as low as possible. Let *m*_*k*_ be the mean of cluster *c*_*k*_. The squared error between a cluster center and its members is defined as:

1 $$ J(c_{k}) = \sum\limits_{x_{i} \in c_{k}} || x_{i} - m_{k}||^{2} $$

K-means seeks to minimize the sum of the squared errors:

2 $$ J(C) = \sum\limits_{k=1}^{K} \sum\limits_{x_{i} \in c_{k}} || x_{i} - m_{k}||^{2}  $$

where *C* is the set of clusters. To minimize Eq. 2, the following algorithm is applied:

1. Randomly assign *K* cluster centers and repeat step 2 and 3.
2. Assign each data point to the closest cluster center.
3. Calculate the new cluster centers.

However, K-means requires the number of clusters to be known. Hence, we applied the elbow method to determine the optimal number of clusters for which the obtained partition is compact, i.e., low *J*(*C*). Naturally, adding more clusters would result in an even more compact partition which may lead to over-fitting. Hence, the variation of *J*(*C*) with respect to *K* would exhibit first a sharp decrease followed by a slow one. The elbow method recommends selecting the number of clusters corresponding to the elbow of the curve *J*(*C*) vs. *K*.

### Bi-LSTM for COVID-19 cumulative cases prediction

After applying K-means, we collect the multivariate time series data for the countries of each cluster to train a prediction model using a Bi-LSTM deep neural network. The motivation is to strengthen the prediction accuracy by forcing the network to train not only on past data to predict the future but also to train it on the future data to predict the past.

#### Multivariate time series data

The multivariate time series have more than one time-dependent variable. Intrinsically, these variables are also dependent on each other. Indeed, it is confirmed that lockdown measures significantly impact the variation of the cumulative number of COVID-19 cases. Our times series consists of:

- Cumulative COVID-19 cases per day. These data are widely available and several APIs provided by government agencies can be queried for this information. We collect data from February 15th to December 31st.
- School closing: This time series describe the level of lockdown imposed on schools where 0 indicates no measures, 1 recommends closing, 2 requires closing (only some levels or categories, e.g., just high school, or just public schools), and 3 requires closing all levels.
- Workplace closing, where 0 indicates no measures,1 recommends closing (or recommend work from home), 2 requires closing (or work from home) for some sectors or categories of workers, and 3 requires closing (or work from home) for all-but-essential workplaces (e.g., grocery stores, doctors).
- Restrictions on gatherings: where 0 indicates no restrictions, 1—restrictions on huge gatherings (the limit is above 1000 people), 2—restrictions on gatherings between 101 and 1000 people, 3—restrictions on gatherings between 11 and 100 people, and 4—restrictions on gatherings of 10 people or less.
- Public transport shutdown where 0 indicates no measures, 1 recommends closing (or significantly reduces volume/route/means of transport available) and 2 requires closing (or prohibit most citizens from using it)
- International travel controls where 0 indicates no restrictions, 1—screening arrivals, 2—quarantine arrivals from some or all regions, 3—ban arrivals from some regions, and 4—ban on all regions or total border closure.

#### Training the prediction model for COVID-19 cumulative cases

**Fig. 2:**
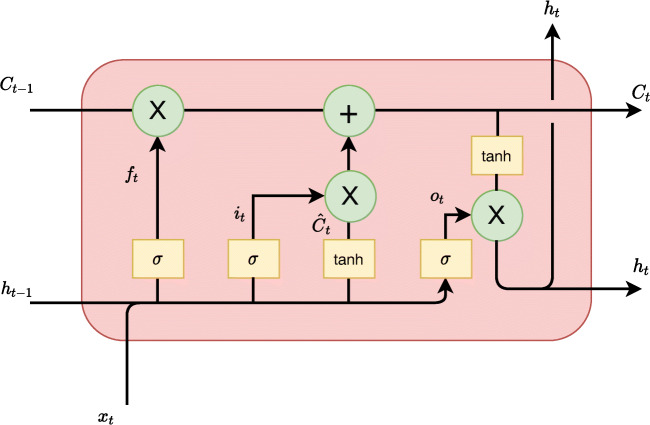
Long Short-Term Memory (LSTM) cell

The building block of the network is the LSTM cell depicted in Fig. 2. Given the current value *x*_*t*_, the previous hidden state *h*_*t*− 1_ and the previous state *C*_*t*− 1_, the following transformations are applied:

3 $$ f_{t} = \sigma\left( W_{f}\cdot [h_{t-1}, x_{t}] + b_{f} \right) $$

4 $$ i_{t}=\sigma\left( W_{i}[h_{t-1},x_{t}] + b_{i}\right) $$

5 $$ \hat{C}_{t}=tanh\left( W_{C}[h_{t-1},x_{t}] +b_{c}\right) $$

6 $$ C_{t}=f_{t}*C_{t-1}+i_{t}*\hat{C}_{t} $$

7 $$ o_{t} = \sigma\left( W_{o}[h_{t-1},x_{t}]+b_{o}\right) $$

8 $$ h_{t} = o_{t}*tanh(C_{t}) $$

where *σ* and *tanh* are the sigmoid and hyperbolic tangent functions, respectively. *f*_*t*_ is the forget gate, *i*_*t*_ is the input gate, and *o*_*t*_ is the output gate. *W* and *b* are the weight matrix and bias vector, respectively. [⋅,⋅] is the concatenation operator, and ∗ is the dot product.

**Fig. 3:**
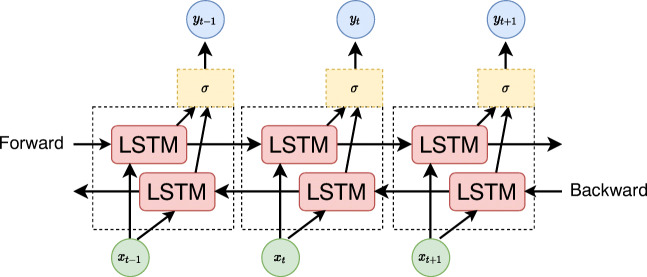
Unfolded architecture of Bidirectional LSTM

Hence, an LSTM layer consists of a sequence of LSTM cells, and the sequence data are fed in a forward way. Bi-LSTM includes another LSTM layer for which the data are fed backward, as depicted in Fig. 3. By stacking multiple Bi-LSTM layers, i.e., feeding the output of one layer to the next one, a deep neural network can be trained to forecast the next day’s cumulative number of cases. The network is trained using backpropagation (Rumelhart et al. 1986; Goodfellow et al. 2016) algorithm to minimize the mean squared error between the actual daily cumulative cases and the value predicted by the network.

## Experiments

The proposed technique is versatile. Indeed, the forecast can be applied to any country. In our experiment, we aim at using information from the previous 6 days to predict the next day’s cumulative cases. We focus on Qatar as a use-case, and we analyze and assess the forecast performance of the proposed technique and compare against multiple techniques with multiple scenarios.

### Evaluation approach

We analyze the performance by:

- Comparing the prediction performance of the proposed approach against LSTM model. We show the benefit of the following: 1—training the learning models on data from all countries in the same cluster. 2—including lockdown information in the training data.
- Comparing against state-of-art techniques including ARIMA, simple moving average with 6-day window and double exponential moving average.
- Evaluating the prediction accuracy by reporting the root mean square error (RMSE), mean absolute error (MAE), coefficient of residual mass (CRM), and the determination coefficient R^2^ where:
  9 $$ RMSE = \sqrt{\frac{1}{n} \sum\limits_{i=1}^{n} (x_{i} -y_{i})^{2}} $$
  10 $$ MAE = \frac{1}{n} \sum\limits_{i=1}^{n} \left| \frac{x_{i}-y_{i}}{x_{i}} \right| $$
  11 $$ CRM = \frac{\sum\limits_{i=1}^{n} y_{i} - \sum\limits_{i=1}^{n} x_{i}}{\sum\limits_{i=1}^{n} y_{i}} $$
  12 $$ R^{2} = \frac{\left( \sum\limits_{i=1}^{n}(x_{i}-\hat{x})(y_{i}-\hat{y}) \right)^{2}}{\sum\limits_{i=1}^{n} (x_{i}-\hat{x})^{2} \sum\limits_{i=1}^{n} (y_{i}-\hat{y})^{2}} $$
  where *x*_*i*_, *y*_*i*_, $\hat {x}$, and $\hat {y}$ are the actual reported cumulative cases, predicted cumulative cases, the average reported cumulative cases, and average predicted cumulative cases, respectively. The best prediction is the one achieving the lowest RMSE, MAE, the highest R^2^, and the closest CRM value to zero.

### COVID-19 in Qatar

**Fig. 4:**
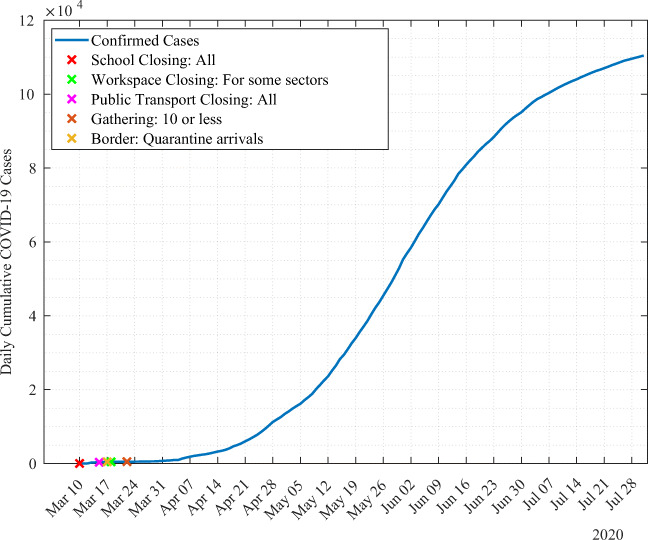
Cumulative COVID-19 cases in Qatar with lockdown measures

We illustrate in Fig. 4 the variation of the daily cumulative cases in Qatar from March 10 to July 31, 2020. Until the end of March, the cumulative cases evolved in a linear trend. Then, numbers have started to grow exponentially until mid-June. By mid-June, the growth of the number of cases has started to slow down. The first confirmed case has been reported on February 29. By July 31, 235 cases have been reported. All lockdown measures have been imposed in March. Schools were all closed on March 10. Then, all public transport services have been shutdown on March 15. Borders have been closed on March 17, and quarantine is required on arrivals from all regions for nationals. Workplace have been also closed for some sectors on March 18 and public gathering for more than 10 persons has been prohibited on March 22. By July 31, the total cumulative cases reached 110,460.

### Data clustering

**Fig. 5:**
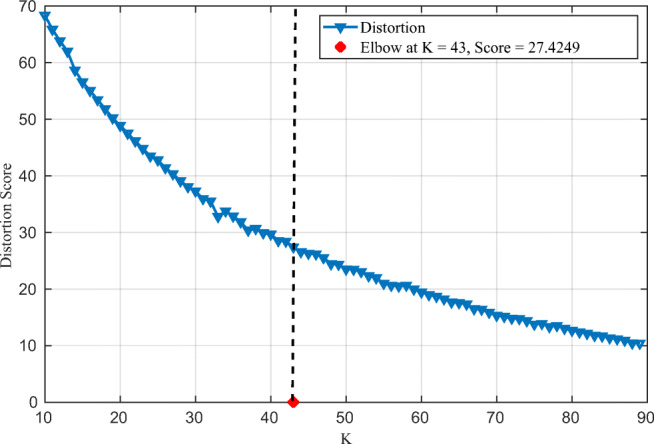
Distortion score for different numbers of clusters. Elbow corresponds to *K* = 43

By clustering the socioeconomic and demographic properties data and the health sector indicators data, we intend to discover countries having similar properties. For the elbow method, we use the distortion, i.e., the mean squared distances to the cluster centers, as a metric. Results are depicted in Fig. 5. The findings suggest that K = 43 corresponds to the elbow and is the optimal number of clusters. Clustering results using K-means show that Qatar shares similar properties as Oman, Bahrain, and United Arab Emirates (UAE). Our findings also show that, for example, Belgium, Canada, Finland, Sweden, and the UK are in the same group.

**Fig. 6:**
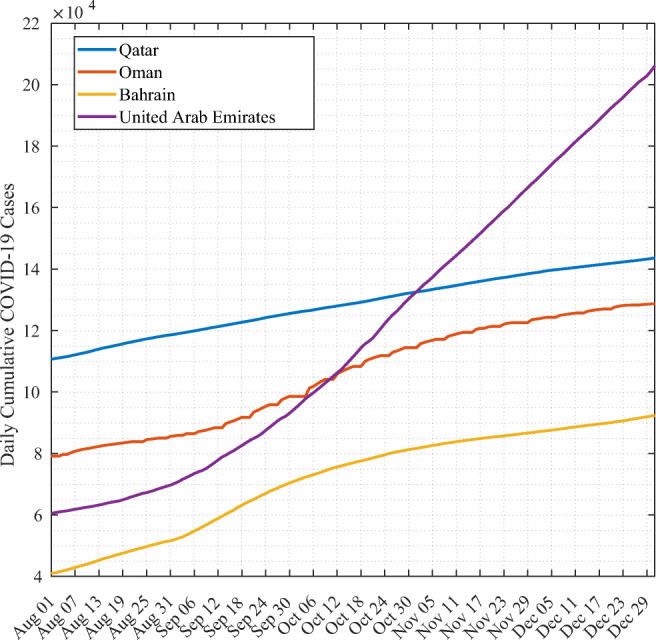
Cumulative COVID-19 cases of countries having similar demographic and socioeconomic properties

Figure 6 shows the cumulative cases of Qatar, Oman, Bahrain, and UAE from August until December 2020. We notice that UAE exhibits the most severe growth in the number of cases, with an exponential-like shape. Oman exhibits two linear trends. The first one, witnessed until mid-September, is linear with slow growth. Then, we notice a second linear trend with a slightly sharper increase in the number of cases. We also notice a similar trend for Bahrain. During the same period, Qatar had the highest reported number of cumulative cases until the end of October, with an overall linear trend throughout this period.

**Fig. 7:**
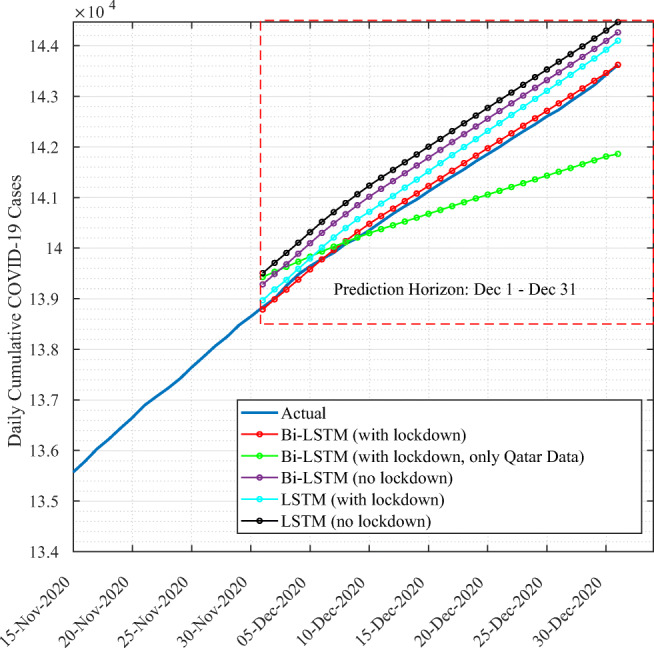
Forecasting results for Qatar using Bi-LSTM vs. LSTM models trained on Qatar cluster data

We illustrate in Fig. 7 the actual growth of number of cases for Qatar and the forecasting results for LSTM and Bi-LSTM with and without lockdown information. Models are trained on data of all countries in the cluster.

For illustration purposes, we show data from November 15 to December 31. The findings show that deep learning techniques succeeded in capturing the trend of cumulative cases in Qatar. The predictions curves are similar to the actual cumulative cases data. To further assess the prediction performance, we conduct quantitative analysis, detailed in Table 1. Results show that Bi-LSTM with lockdown information achieved the lowest RMSE, MAE, the highest R^2^ score, and the closest CRM value to zero while the best performance is achieved when the model is trained on all data of the cluster to which Qatar belongs rather than Qatar data only. In fact, this is confirmed by both the RMSE and CRM values comparison. Results also allow us to confirm the importance of including lockdown information as they improved the performance of both LSTM and Bi-LSTM models.

**Table 1:** Evaluation results of deep learning models

	RMSE	MAE	R^2^	CRM
Bi-LSTM with lockdown	245.1	176.02	0.996	− 0.0003
Bi-LSTM with lockdown (only Qatar data)	258.24	175.22	0.996	− 0.0016
Bi-LSTM without lockdown	389.6	321.9	0.981	− 0.00065
LSTM with lockdown	373.03	325.6	0.99	− 0.00061
LSTM without lockdown	380.19	349.03	0.977	0.0071

**Fig. 8:**
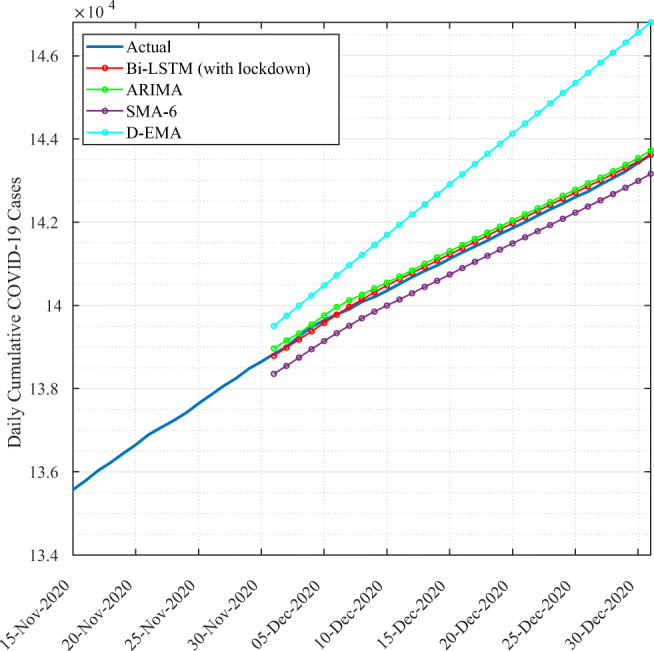
Forecasting results for Qatar using Bi-LSTM with lockdown compared to state-art time series forecasting approaches

We further compare the proposed approach against state-of-art time series forecasting approaches, including ARIMA, Simple Moving Average with 6-day window (SMA-6), and Double Exponential Moving Average (D-EXP-EMA). Figure 8 illustrates the forecasting results. It clearly shows how the proposed technique outperformed other approaches. In fact, SMA-6 and ARIMA tend to underestimate the total number of cases, while D-EMA overestimates the number of cases. This performance is quantitatively confirmed by the evaluation metrics detailed in Table 2

**Table 2:** Performance evaluation of Bi-LSTM with lockdown, ARIMA, SMA-6, and D-EXP-MA

	RMSE	MAE	R^2^	CRM
Bi-LSTM with lockdown	245.1	176.02	0.996	− 0.0003
ARIMA	2109.1	2099.84	0.744	− 0.02
SMA-6	1356.5	1287.4	0.89	− 0.012
D-EXP-MA	2110.2	1562.7	0.744	0.01

### Discussion

Predicting cumulative COVID-19 cases is a challenging task as it depends on several complex and highly dependable parameters. The disease outbreak heavily relies on, among others, the lockdown measures and how fast they are imposed. In our proposed approach, we aimed at incorporating several parameters to achieve accurate forecast by the following: 1—maximizing the data used to train a forecasting model by grouping countries having similar properties: 2—using a Bi-LSTM model trained on both numbers of cases and lockdown measures. It has been confirmed that rushing towards easing lockdown measures has contributed to an increase in the number of cases. This has been the case in Florida and Texas, USA. In fact, Florida reopened specific businesses on May 4, 2020, and Florida Keys businesses were allowed to reopen to visitors on June 1, 2020. In Texas, school districts were allowed to open. Both states witnessed significant growth in the number of cases. The proposed solution may assist decision-makers in putting future short-term plans to overcome the epidemic and carefully choose the opening strategy.

## Conclusion

COVID-19 outbreak has reshaped the whole world and tested the readiness of the countries to a sudden health crisis. It is of paramount importance to address this emergency with multidisciplinary collaborations at the level of local communities, states, and countries with spirit of sharing and transparency. Data science and machine learning techniques are potential technologies that can hugely contribute to addressing these unprecedented challenges in modern history. In this research effort, we proposed a deep learning–based approach to forecast the daily cumulative COVID-19 cases. Countries having similar demographic socioeconomic and health sector properties are clustered together in order to train the forecasting model on data associated to the cluster rather than data of each country separately. The findings showed that Qatar has similar demographic, socioeconomic, and health sector properties as UAE, Bahrain, and Oman. Using Bi-LSTM and including lockdown information in the forecasting data, the proposed approach achieved significant improvement in the prediction performance compared to state-of-art techniques with Qatar as a use case. This is confirmed through quantitative analysis using the root mean square error, mean absolute error, coefficient of residual mass, and the determinant coefficient.

In future work, we will establish lockdown-easing scenarios and investigate the forecasting results to analyze the impact of the easing on the increase/decrease of the number of cumulative cases. In addition, as the vaccination campaign started worldwide, we will address its impact on the COVID-19 outbreak in Qatar, where the first batch of Pfizer-BioNTech vaccine arrived on December 21, 2020, using data science and analytics approaches.

## Footnotes

1 https://ourworldindata.org/covid-vaccinations
